# Potential of Plasma Rich in Growth Factors (PRGF-Endoret) to Enhance the Efficacy of Assisted Reproductive Techniques in Refractory Cases

**DOI:** 10.7759/cureus.26623

**Published:** 2022-07-06

**Authors:** Jon Ander Agirregoikoa, Jose Luis de Pablo, Maria de la Fuente, Eduardo Anitua

**Affiliations:** 1 Embryology Laboratory, Clínica ART, Vitoria, ESP; 2 Regenerative Medicine Laboratory, Biotechnology Institute ImasD, Vitoria, ESP; 3 Regenerative Medicine Laboratory, Biotechnology Institute ImasD, Instituto Eduardo Anitua, Vitoria, ESP

**Keywords:** platelet-rich plasma, infertility, implantation, blastocyst, assisted reproductive techniques

## Abstract

Aim: Nowadays, infertility problems affect a high percentage of couples. This study aimed to evaluate the effect of plasma rich in growth factors (PRGF-Endoret, hereafter PRGF) as a promising coadjuvant therapy in assisted reproductive techniques and its possible role in implantation and pregnancy rates. This retrospective study included 36 PRGF cycles in 27 women with one of the following reproductive disorders: recurrent implantation failure (n = 16), repeated abortion (n = 8), and thin endometrium (n = 3).

Methods: PRGF was obtained from each patient and administered as three consecutive intrauterine instillations. The endometrial thickness was measured after each PRGF infusion and a good-quality embryo transfer was performed for every patient. Endometrial thickness, biochemical pregnancy, and miscarriage rate were the primary measured outcomes.

Results: PRGF increased the endometrial growth respecting the initial thickness in all cases. The biochemical pregnancy rate determined as positive beta-human chorionic gonadotropin (β-hCG) was 59%, considering the total number of patients; the ongoing pregnancy percentage was 48%. PRGF application day was relevant with a significant probability of achieving pregnancy (p < 0.01) when the first PRGF infusion was carried out beyond 6.5 days after the first day of the woman's cycle and the second one beyond 9.5 days of the menstrual cycle.

Conclusions: Intrauterine autologous PRGF infusion is a safe, easily accessible, and inexpensive therapy that could collaborate in fertility treatments by optimizing the endometrium for implantation and thus favoring the crosstalk between the embryo and the uterus improving the embryo-maternal dialogue.

## Introduction

Infertility is defined as the inability to conceive within 12 months with unprotected intercourse for at least a year. In Spain, infertility affects 17% of couples of childbearing age who have trouble getting pregnant or successful delivery. Assisted reproductive techniques (ART) have presented important advances in recent years, with 9% of babies born by these methods.

Implantation is crucial in reproduction [1,2], and a failure means pregnancy loss. In reproductive medicine, recurrent implantation failure (RIF) is the condition resulting from repetitive unsuccessful cycles of IVF or intracytoplasmic sperm injection (ICSI) with the absence of implantation after repeated good-quality embryo transfers [3]. Repeated abortion (RA) or recurrent miscarriage is the occurrence of two or more consecutive pregnancy losses before 20-24 weeks of gestation, including micro-abortions, those in which after a positive pregnancy test, bleeding and gestational loss occur within a few days [4].

In this sense, assisted reproduction techniques should be directed toward the improvement of implantation, and promote the dialogue between the embryo and the endometrium that starts even before implantation. Also, the embryo-maternal crosstalk should be enhanced, favoring the processes of apposition, adhesion, and implantation [5]. In clinical practice, appropriate endometrium thickness suggests an optimal endometrial growth and receptivity that acts as a biosensor for embryo quality; although pregnancies have been reported at 4 and 5 mm, it is noticeable that an endometrial thickness less than 6 mm is associated with a lower probability of pregnancy [6]. Endometrial receptivity is crucial for successful embryo implantation since suboptimal endometrial growth or vascularity might lead to repeated cycle cancellations or RIF. Indeed, implantation is a multifactorial and dynamic process controlled by several complex molecules that involve coordinated effects and crosstalk of autocrine, paracrine, and endocrine factors, playing a crucial role in preparing receptive endometrium and blastocyst [7,8].

Recently, the use of platelet-rich plasma (PRP) has arisen as an interesting option in RIF and thin endometrium (TE). Plasma rich in growth factors (PRGF) technology is an autologous biological approach based on platelet-enriched plasma free of leucocytes. After platelet activation, a myriad of active growth factors, proteins, and cytokines present in the alpha granules within platelets are released to the surrounding environment, triggering several biological cellular processes. PRGF is a versatile technology with proven effects and great potential in several medical fields in regenerative medicine [9]. In endometrial fibroblasts, PRGF improves their biological activity in vitro, enhancing the regulation of several cellular processes implied in endometrial regeneration [10].

Several studies have concluded that PRP is an effective treatment for TE, expanding the thickness, and in RIFs, where the application of intrauterine PRP as an adjuvant treatment could help improve the pregnancy rate [11-16].

In this retrospective observational study, the effect of the application of PRGF in women with different infertility etiology is described. PRGF instillation was applied to patients suffering from RIF, recurrent miscarriage, or those whose endometrium failed to reach optimal thickness. The purpose was to investigate the use of PRGF for improving implantation and pregnancy rate in patients with a previous history of infertility.

## Materials and methods

Patients

This retrospective observational study was conducted at the ART Clinic, a private human reproduction center located in Vitoria, Spain. Data were collected from an anonymized database from 2019 to 2020. A total of 36 cycles in 27 patients were analyzed. All participating women were properly informed about the intervention and gave informed consent for the application of PRGF. The age group of the women included in the study was 34-47 years. They were divided into three groups: recurrent implantation failure (RIF), i.e., no pregnancy after two or more good quality embryos were transferred; repeated abortion (RA), i.e., two or more miscarriages after good quality embryos; and thin endometrium (TE), i.e., less than 6 mm endometrium after hormone replacement therapy (HRT). All patients were treated with HRT protocol as usual in the clinical practice for endometrial preparation, administering E2 valerate orally.

PRGF preparation and application

For PRGF obtention, 18 ml of venous blood was drawn from the patients in sodium citrate tubes. Tubes were centrifuged at 580 g for eight minutes at room temperature in a PRGF system centrifuge (BTI Biotechnology Institute, S.L., Álava, Spain). The whole plasma column was aspirated avoiding the buffy coat containing the leucocytes. After the addition of the PRGF activator (calcium chloride), the released supernatant was collected for the first instillation or stored frozen for posterior applications.

Approximately one week after estrogen administration, 1 ml PRGF was instilled into the intrauterine cavity under ultrasound guidance with an intrauterine insemination (IUI) cannula. After 48-72 hours, the second instillation was carried out and 48-72 hours later, the third instillation was done. Endometrial thickness was measured before each instillation; every patient received three PRGF applications regardless of whether the endometrial thickness was 7 mm or less. PRGF cycle was performed once or more times depending on the patient, understanding a cycle as three consecutive instillations.

Embryo transfer

Embryo transfer was performed in a cycle of vitrified embryos (good quality own or egg donation embryos) with substituted hormonal therapy. Endometrial preparation begins on the second day of menstruation with 4-6 mg/day of estradiol valerate (Progynova; Bayer Hispania, S.L., Sant Joan Despí, Spain). The first ultrasound is performed 5-11 days after starting the medication and then every 48-72 hours. Together with ultrasound, intracavitary instillation of PRGF is performed. After performing the third inoculation, luteal phase support is started with 200 mg/eight hours of micronized progesterone (Utrogestan; SEID S.A., Barcelona, Spain). After the transfer of a single blastocyst, the medication is maintained until the serum levels of beta-human chorionic gonadotropin (β-hCG) measurement are performed (12 days after the embryo transfer), and if this is positive until the 12th week of pregnancy. The primary studied outcomes were endometrial thickness, pregnancy rate, and miscarriage rate.

Statistical analysis

Data collected were analyzed using SPSS 15.0 (SPSS Inc., Chicago, IL) for statistical analysis. Descriptive statistics were performed using absolute and relative frequency distributions for qualitative variables and mean values and standard deviations analyses for quantitative variables. The chi-square test was used to compare the qualitative variables and Student's t-test for quantitative variables. A p-value of <0.05 was established as a statistical significance level.

## Results

Table 1 shows individual data of the 27 women included in the study including all relevant information. More specifically, Table 2 collects the data of all the patients classified according to the indication (RIF, RA, or TE).

**Table 1:** Individual data of the 27 women involved in this study All patients were recruited in 2019 and 2020 at ART Clinic in Vitoria, Spain. The day of each PRGF application is counted from the beginning of the menstrual cycle. A biochemical pregnancy implies a positive β-hCG and an ongoing pregnancy is defined as a pregnancy with a detectable heart rate at 12 weeks of gestation. PRGF: plasma rich in growth factors; RIF: recurrent implantation failure; RA: repeated abortion; TE: thin endometrium; β-hCG: beta-human chorionic gonadotropin.

Patient	Age (years)	Indication	Number of PRGF cycles	1^st^ PRGF application (day of the menstrual cycle)	Endometrial thickness (mm) (1^st^ control)	2^nd^ PRGF application (day of the menstrual cycle)	Endometrial thickness (mm) (2^nd^ control)	3^rd^ PRGF application (day of the menstrual cycle)	Endometrial thickness (mm) (3^rd^ control)	Biochemical pregnancy	Ongoing pregnancy
1	32	RIF	1	7	5	9	7.6	11	8.6	Negative	-
2	38	RIF	1	6	6.8	9	8.0	11	9	Positive	Yes
3	40	RIF	1	10	7.5	12	7.5	14	7.5	Positive	Yes
4	39	RIF	1	11	7.4	14	9.2	17	9.3	Positive	Yes
5	42	RIF	1	5	8	7	9.3	10	9.8	Negative	--
6	40	RIF	1	5	5.2	7	6.8	10	8.2	Positive	Yes
7	34	RIF	1	5	8.7	7	9.2	9	10.3	Positive	No
8	40	RIF	2	5	6.2	8	6.5	10	8	Negative	-
9	47	RIF	1	5	7.8	10	10.3	12	14.1	Negative	-
10	38	RIF	1	7	5.4	9	5.8	11	6.8	Positive	Yes
11	44	RIF	2	8	6	10	7.8	12	-	Positive	Yes
12	43	RIF	1	9	7.8	11	7.8	13	7.8	Positive	Yes
13	43	RIF	2	5	6.1	7	7.8	11	10.4	Positive	Yes
14	37	RIF	1	6	8.50	8	9.2	10	9.2	Negative	-
15	38	RIF	1	6	7.7	8	7.9	9	7.9	Negative	-
16	40	RIF	1	5	4.5	7	5.6	10	8.5	Positive	No
17	42	RA	3	5	6.2	7	6.9	9	7.6	Negative	-
18	44	RA	1	4	5.6	6	7.6	9	8.2	Negative	-
19	41	RA	1	5	6	7	7.5	10	10	Positive	Yes
20	36	RA	1	7	6.9	9	7.6	11	8.3	Positive	No
21	35	RA	1	5	6.7	7	6.9	9	6.9	Positive	Yes
22	39	RA	1	5	5.2	8	6.0	10	7	Negative	-
23	47	RA	1	7	5.5	10	6.6	12	8	Positive	Yes
24	34	RA	3	5	5.3	8	6.5	11	6.5	Negative	-
25	35	TE	1	11	4.7	13	5.5	15	6.4	Positive	Yes
26	37	TE	1	5	4.3	8	6.2	10	6.4	Positive	Yes
27	37	TE	1	5	6.1	7	7.0	10	8	Negative	-

**Table 2:** Patients' characteristics Table summarizing patients' characteristics distributed according to the indication (reproductive disorder). β-hCG: beta-human chorionic gonadotropin.

Indication	Recurrent implantation failure (RIF)	Repeated abortion (RA)	Thin endometrium (TE)
Age	40 ± 4	40 ± 5	36 ± 1
Number of patients, n	16	8	3
Endometrium thickness (mm) (mean ± SD)	6.3 ± 1.3	7.4 ± 1.2	8.4 ± 1.6
1^st^ application (mm)	6.8 ± 1.3	5.9 ± 0.6	5.0 ± 0.9
2^nd^ application (mm)	7.9 ± 1.3	7.0 ± 0.6	6.2 ± 0.8
3^rd^ application (mm)	9.0 ± 1.7	7.8 ± 1.1	6.9 ± 0.9
Number of positive β-hCG, n (% of total)	10 (63%)	4 (50%)	2 (67%)
Number of ongoing pregnancies, n (% of total)	8 (50%)	3 (38%)	2 (67%)
Miscarriages, n (%)	2 (13%)	1 (13%)	0 (0%)

The average age of the women participating in the study was 39 years old (range 34-47 years). They were divided into three groups: RIF (n = 18), RA (n = 14), or TE (n = 4). Out of the 36 cycles realized, 28 were single PRGF cycles (78% of the cycles with a 52% of pregnancy rate), six consisted of two cycles (17% of the PRGF cycles representing 33% positive β-hCG), and two cases (5%) consisted of three cycles, unfortunately, with no pregnancies.

**Figure 1:**
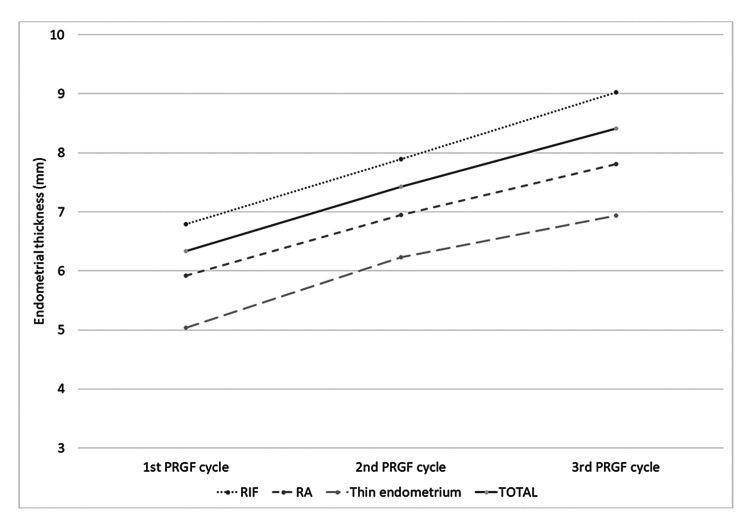
Endometrial thickness Endometrial thickness progression. The endometrium thickness (in mm) after each PRGF application was measured with ultrasound. The graph shows the variation in each group (RIF or recurrent implantation failure, RA or repeated abortion, and TE or thin endometrium) and in the total number of patients. PRGF: plasma rich in growth factors; RIF: recurrent implantation failure; RA: repeated abortion.

Regarding endometrium thickness, for all 27 patients analyzed, the mean measure after the first PRGF application was 6.3 ± 1.3 mm; this value increased to 7.4 ± 1.2 mm after the second PRGF instillation and to 8.4 ± 1.6 mm after the third PRGF infusion. Figure 1 shows endometrial thickness variation by indication (RIF, RA, or TE) after each PRGF application. In every case, there is an increase in endometrial width.

The biochemical pregnancy rate was 59%, considering the total number of patients (16 positive β-hCG out of 27 women); by groups, the percentages of biochemical pregnancy were 63% in the RIF group (10 out of the total women), 50% in RA (four positives in eight cases), and 67% in women with a TE (two out of three women). The ongoing pregnancy rate was 48%, considering that 13 out of the 27 women had a gestational sac; from these, the success rate was 50% for RIF (eight out of 16 women), 38% in the recurrent abortion group (three out of eight women), and 67% (two out of three women with TE) in women with TE. Unfortunately, of the total β-hCG positives, three women had an abortion.

The statistical analysis showed that the day of PRGF application was a relevant fact, and there was a statistically significant probability of achieving pregnancy (p < 0.01) when the first PRGF infusion was carried out beyond 6.5 days after the first day of the woman's cycle. Likewise, there was also a statistically significant probability of getting pregnant (p < 0.05) when the second instillation with PRGF was performed beyond 9.5 days after the first day of the women's menstrual cycle.

## Discussion

PRGF is a pioneering autologous PRP technology. It has been shown to improve regeneration in various tissues through the action of a myriad of growth factors, hormones, and cytokines that are released locally and in a sustained way due to its behavior as a natural reservoir. PRGF is a type of leukocyte-free PRP with a moderate and optimal platelet concentration; its clinical efficacy has been demonstrated in several medical fields [17]. Recently, the beneficial effects of PRP have been reported in gynecology, obstetrics, and reproductive medicine [18-20] as an interesting therapeutic tool with minimal risks for disease transmission and immunogenic and allergic responses due to its autologous source.

Just after fertilization, the developing embryo needs to be embedded within the endometrium; both the embryo and uterus require the secretion and suppression of specific proteins promoting implantation, including the expression of adhesion molecules on the cell surface, secretion of growth factors, and morphologic cell differentiation. A mixture of important proteins is necessary for uterine preparation, including the induction of a variety of cytokines and growth factors that help to stimulate uterine lining proliferation and endothelial growth and contribute to a completely thickened endometrium. These growth factors include transforming growth factors (TGF family) that may play a role in protecting the endometrium from extensive fibrosis and scarring, platelet-derived growth factor (PDGF) that promotes endometrial tissue repair, and epidermal growth factor (EGF) and its receptors that are important for implantation and embryo development; vascular endothelial growth factor (VEGF) aids in the development and thickening of the endometrium, mediating angiogenic activity and playing multifaceted roles in embryo implantation [7,21,22].

All the growth factors and cytokines mentioned and many more are present among the innumerable molecules contained in the PRGF. The beneficial effects of the PRP over human endometrial fibroblasts in vitro have been recently tested, promoting endometrial regeneration [10,23,24].

In this study, 27 women were enrolled to investigate the clinical effect of PRGF on several reproductive failures; they belonged to three groups: RIF, repeated pregnancy loss, and TE. Endometrial receptivity is a key factor for successful embryo implantation and pregnancy. Thus, the endometrial factor should be considered as the main target in the search for a tool for reproductive disorders.

The therapeutic strategy in RIF cases was conducted at solving a possible endometrial problem, that is, considering an “endometrial factor” as the cause of the failure in the implantation process. RIF patients receiving PRGF infusions increased the percentage of pregnancy rate in a very appreciable manner, achieving 10 out of 16 positive β-hCG pregnancies. In women with repeated abortion, the pregnancy rate was four out of eight patients, indicating a hopeful solution for this disorder. The number of patients with TE was quite insufficient, but despite this, pregnancy was achieved in two out of three cases; it should be noted that positive β-hCG was detected with endometrial thickness under 7 mm. In all cases, PRGF increased the endometrial growth respecting the initial thickness.

The cellular processes that are involved in the improvement of embryo implantation and pregnancy after PRGF treatment in reproductive disorders are not yet well known; probably, it should be a combination of the effect over multiple cellular pathways involving induction of the cell proliferation, chemotaxis, regeneration, extracellular matrix synthesis, remodeling, improving vascularity, and epithelialization. The implantation process is a consequence of an inflammatory and anti-inflammatory equilibrium and an imbalance could lead to cases of RIF [25]. PRP has demonstrated anti-inflammatory action in several medical fields, namely, PRGF-Endoret [26]. The positive effects of PRP on implantation and gestation should occur via its anti-inflammatory molecules in the case of undetected or silent endometritis and even working over a possible underlying infection, thanks to the anti-microbial role of PRPs [27,28].

Precisely, in clinical practice, the positive effect of PRP infusion on pregnancy achievement has been widely proven, improving reproductive outcomes of women with TE or RIF [29,30].

The results of this study show that the day of PRGF application seems to be a crucial fact; based on this analysis, the probability of achieving pregnancy is higher if the gynecologists wait until the seventh day of the menstrual cycle to do the first PRGF infusion or until the 10th day to instill for the second time. This could serve as a guideline when planning the PRGF instillations.

Although this study has several limitations such as the small study population in TE, it could be considered a good starting point for future research. Prospective analysis, randomized controlled clinical trials, and study of outcome in terms of live birth rates are needed to confirm our observations.

## Conclusions

In conclusion, our data indicate that intrauterine autologous PRGF infusion is an easily accessible and inexpensive therapy that could collaborate in fertility treatments by preparing the endometrium for implantation and thus favoring the crosstalk between the embryo and the uterus. It is a safe autologous coadjuvant treatment for optimizing endometrium, especially in patients with RIF history.
